# 

**Published:** 1864-12

**Authors:** 


*Contents.*

ORIGINAL COMMUNICATIONS........................................ 201

1. Dry-Cupping as an Excito-Secretory Remedial Agent, by B. H. Washington, Acting Surgeon. 2. Loss of the Pericranium and its Reformation without Necrosis or Exfoliation of the Cranial Bones, by Le Grand Capers, Surgeon P. A. C. S. 3. Report of a Case of Acute Laryngitis, in which Laryngo-Tracheotomy was performed, by Ignatius D Thompson, Assistant-Surgeon P. A. C. S. 4 The Position of the Hand in Fracture of the Fore-arm, by Paul F. Eve, M. D., Augusta, Ga. 5. A Case of Snake Bite successfully treated with Calomel and Iodide of Potassium, by J. J. Knott, Assistant-Surgeon Fifty-Third Georgia Regiment.

CHRONICLE OF MEDICAL SCIENCE............................ 213

Notes upon Diptheria, by W. F. Wade, Senior Physician to the Queen's Hospital, Birmingham. Is Alcohol Food? by Thomas Inman, M. D. The Vessels Concerned in the Production of Phlegmasia Dolens. On Dislocations of the Thumb at the Metacarpo-Phalangeal Joint, showing that the difficulty of reducing arises from Malposition of the Tendon of the Long Flexor, by J. C. Wordsworth, Esq., F. R. C. S. Purulent Conjunctivitis in Newly-Born Infants; Methods of Treatment, by Mr. Ernest Hart. Liquor Chlorinæ.

INTERESTING ANALYSES OF ARTICLES IN FOREIGN JOURNALS........................................ 220

Observations on the Operation for the Division of the Ciliary Muscle, by Henry Hancock, F. R. C. S. Alkaloids and Alcohol. Treatment of Chronic Dysentery with Ipecacuanha, by Dr Willshire, of Charing-Cross Hospital. On the Arcus Senilis. Topical Injections of Strychnine. Chylous Urine Successfully Treated by Tincture of Muriate of Iron. What is Carbonic Acid? Extreme Mobility of both Kidneys.

